# Associations of Preoperative and Early Postoperative Biomarkers with Wound Complications in a Clinically Restricted Surgical Population: A Retrospective Cohort Study

**DOI:** 10.3390/jcm15197647

**Published:** 2026-10-02

**Authors:** Emre Erdoğan, Elif Nur Gencer, Teoman Bekir Yeni, Furkan Türkoğlu, Özgür Doğan

**Affiliations:** 1 Department of General Surgery, Aktif International Hospital, 41275 Kocaeli, Türkiye; genelcerrahemreerdogan@gmail.com; 2 Chronic Wound and Burn Unit, Tuzla State Hospital, 34947 Istanbul, Türkiye; elifnurkocmar@yahoo.com; 3 Department of Orthopaedics and Traumatology, Halil Şıvgın Çubuk State Hospital, 06760 Ankara, Türkiye; drteomanbekiryeni@gmail.com; 4 Department of Orthopaedics and Traumatology, Ankara Bilkent City Hospital, 06800 Ankara, Türkiye; dr.ozgurdogan@gmail.com

**Keywords:** postoperative wound complications, wound healing, host biology, perioperative biomarkers, serum sodium, total protein, wound care

## Abstract

**Background/Objectives**: Postoperative wound complications remain a clinically important challenge across surgical disciplines, while the contribution of preoperative biological status and the early postoperative systemic response remain incompletely understood. This study aimed to investigate whether routinely available hematological and biochemical biomarkers measured before surgery and during the early postoperative period were associated with postoperative wound complications. **Methods**: This retrospective single-center study included 58 patients undergoing surgical treatment for peri-ankle fractures, a clinically relevant setting for investigating postoperative wound complications. Demographic characteristics and routine laboratory parameters obtained within 24 h before surgery and during the first 6 postoperative hours were recorded. Patients were classified according to the presence or absence of postoperative WoC. Univariate analyses, Benjamini–Hochberg false-discovery-rate (FDR) adjustment, receiver operating characteristic (ROC) curve analyses, and separate Firth penalized multivariable logistic regression models for preoperative and early postoperative parameters were performed. **Results**: Postoperative WoC developed in 18 patients (31.0%). In the preoperative analysis, hemoglobin, hematocrit, platelet count, sodium, chloride, and total protein differed significantly between groups. Female sex was also significantly associated with WoC. After FDR adjustment, the preoperative platelet count and total protein remained statistically significant. In the early postoperative period, hemoglobin, hematocrit, lymphocyte count, basophil count, sodium, chloride, creatinine, neutrophil-to-lymphocyte ratio (NLR), and prognostic nutritional index (PNI) differed significantly between groups. After FDR adjustment, sodium, basophil count, chloride, hemoglobin, and NLR remained statistically significant. Preoperative total protein showed the highest discriminatory performance (AUC = 0.886, 95% CI 0.797–0.976), whereas postoperative sodium demonstrated the highest early postoperative performance (AUC = 0.808, 95% CI 0.694–0.923). In the preoperative Firth model, female sex (OR = 7.209, 95% CI 1.189–57.415; *p* = 0.031) and higher preoperative total protein (OR = 1.399, 95% CI 1.168–1.788; *p* < 0.001) were associated with WoC. In the early postoperative Firth model, lower serum sodium was associated with postoperative WoC (OR = 0.477, 95% CI 0.241–0.789; *p* = 0.001). **Conclusions**: These findings indicate exploratory associations between routinely available biomarkers and postoperative wound complications. Their clinical utility remains to be established in larger prospective studies.

## 1. Introduction

The restoration of tissue integrity after surgery is essential for successful recovery and depends on an orderly sequence of hemostasis, inflammation, cellular proliferation, and tissue remodeling, all of which must occur in a coordinated manner [1]. Impaired wound healing remains an important clinical challenge across a wide range of surgical specialties, including general, plastic, vascular, and orthopedic surgery. Postoperative wound complications, including wound dehiscence, prolonged wound drainage, and surgical site infection, frequently require additional interventions, delay rehabilitation, prolong hospitalization, and increase healthcare costs [2,3]. Although advances in surgical techniques, perioperative management, and infection prevention have reduced many technical causes of wound failure, patients undergoing comparable procedures often experience markedly different healing outcomes. These observations suggest that surgical technique alone cannot fully explain postoperative wound repair, supporting the hypothesis that host-related biological factors contribute to individual differences in healing outcomes.

Routine hematological and biochemical biomarkers have attracted increasing clinical interest because they provide inexpensive and readily available information about an individual’s inflammatory, immunological, and nutritional status. Several studies across different medical and surgical specialties have demonstrated that composite inflammatory indices derived from routine blood tests are associated with disease severity, postoperative complications, and long-term clinical outcomes [4,5,6]. However, their relevance to postoperative wound healing remains less well established. Existing studies are further limited by substantial heterogeneity in patient populations, surgical procedures, and anatomical regions, making the independent contribution of host-related biological factors difficult to interpret [7,8,9,10]. The clinical relevance of these biomarkers for postoperative wound healing remains incompletely understood, particularly when preoperative biological status and the early postoperative inflammatory response are considered together.

Within this broader surgical context, a clinically restricted population with a well-recognized susceptibility to wound complications may provide a useful model for investigating host-related biological factors while limiting variability arising from anatomy and surgical technique. The ankle region is particularly suitable for this purpose because the distal tibia is covered by a thin soft-tissue envelope with limited muscular coverage, relatively poor vascularity, and minimal soft-tissue reserve. Fracture-related soft-tissue injury, postoperative edema, and surgical dissection may further increase the biological demands of tissue repair, contributing to the occurrence of wound complications despite meticulous surgical technique and perioperative management [2,3,11,12,13]. Thus, this clinically restricted surgical setting provides a relevant model for investigating whether interindividual differences in routinely measurable biological characteristics are associated with postoperative wound outcomes beyond local procedural factors.

Therefore, the present study aimed to investigate whether routinely obtained hematological and biochemical biomarkers are associated with the development of postoperative wound complications. We hypothesized that biomarkers reflecting both the patient’s baseline biological status and the early systemic response to surgical injury would be associated with the risk of postoperative wound complications.

## 2. Materials and Methods

### 2.1. Study Design and Patient Selection

This retrospective cohort study was designed to investigate the association between routinely available preoperative and early postoperative hematological and biochemical biomarkers and the development of postoperative wound complications. The study protocol was approved by the local Ethics Committee (Approval No. E1-22-3072) and was conducted in accordance with the ethical principles of the Declaration of Helsinki. Retrospective data extraction and subsequent statistical analyses were initiated only after Ethics Committee approval had been obtained. This study was designed and reported in accordance with the Strengthening the Reporting of Observational Studies in Epidemiology (STROBE) statement. A completed STROBE checklist has been provided as Supplementary Materials.

The medical records of consecutive adult patients who underwent operative treatment for peri-ankle fractures, defined as distal tibial metaphyseal, isolated malleolar, bimalleolar, trimalleolar, talar, and calcaneal fractures, between January 1 and 30 November 2022, were retrospectively reviewed. Restricting patient inclusion to an 11-month period within a single calendar year was intended to minimize temporal variations in perioperative management, implant selection, surgical experience, operating room conditions, laboratory practices, and institutional clinical care pathways, thereby reducing the potential sources of treatment-related heterogeneity.

Adult patients (≥18 years) who underwent open reduction and internal fixation (ORIF) through a single open surgical approach, with complete clinical, radiological, operative, and laboratory records, were eligible for inclusion. Percutaneous fixation performed in addition to a single open approach was permitted. Thus, patients treated with a lateral open approach combined with percutaneous medial fixation, or vice versa, were included, whereas fractures requiring two or more open surgical approaches were not. The selected fracture types involve anatomically adjacent structures and share relevant clinical and soft-tissue characteristics, allowing them to be evaluated within a clinically defined surgical cohort while limiting, but not eliminating, anatomical heterogeneity.

**Figure 1:**
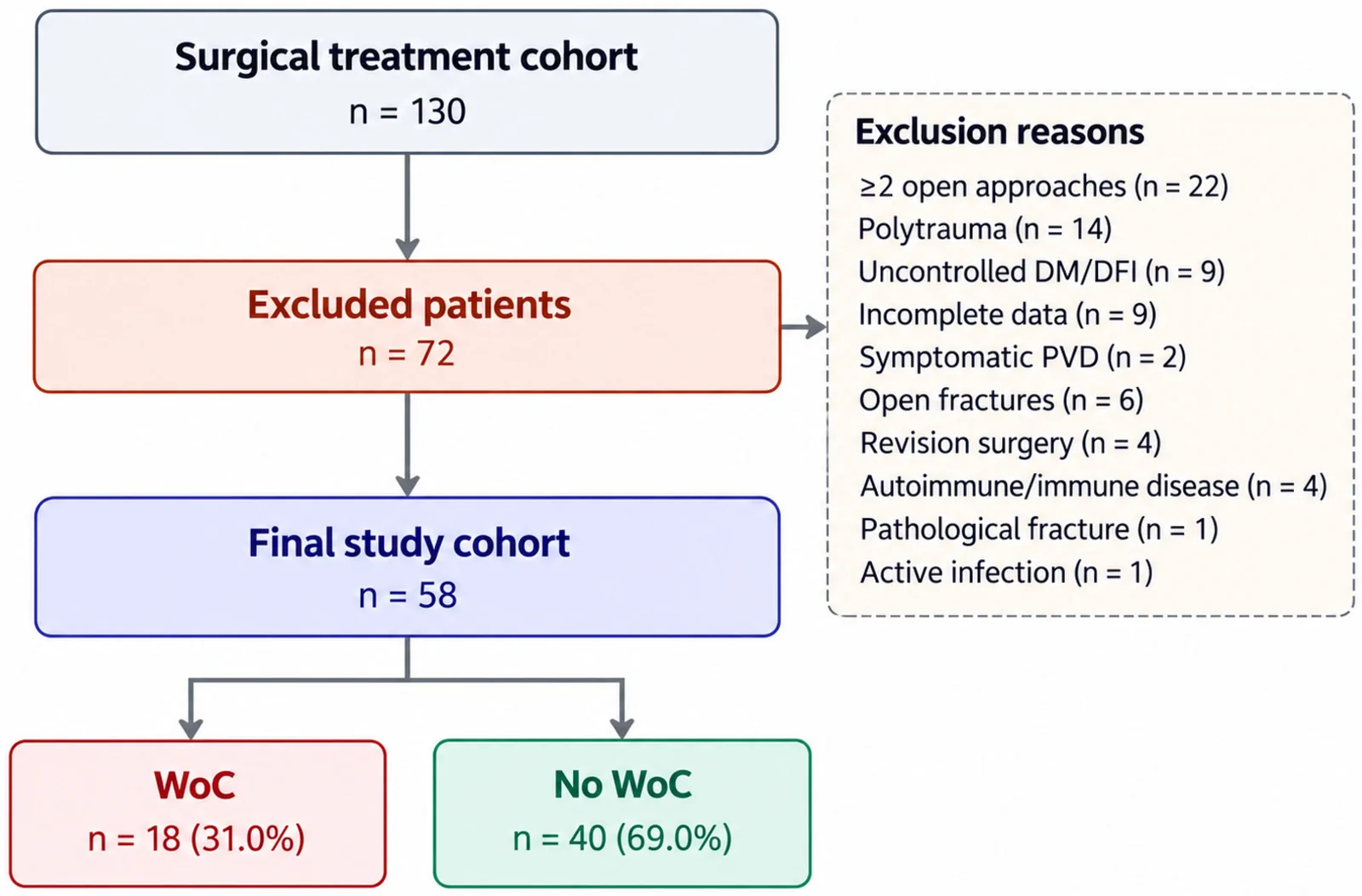
Flow diagram of patient selection, exclusion criteria, and fracture diagnosis distribution in the final study cohort.

Patients treated with closed reduction and internal fixation were excluded because the absence of a surgical incision precluded the assessment of postoperative wound healing. Patients requiring two or more open surgical approaches were also excluded because the extent of surgical exposure is an established independent risk factor for postoperative wound complications and could confound the evaluation of host-related biological factors. Additional exclusion criteria included pathological fractures, open fractures, polytrauma injuries likely to substantially influence the systemic inflammatory response, revision procedures, pre-existing active infection, poorly controlled diabetes mellitus (DM) (HbA1c ≥ 8.0%) or active diabetic foot infection (DFI), chronic inflammatory or autoimmune disorders, immunosuppressive therapy, symptomatic peripheral vascular disease (PVD), and incomplete perioperative laboratory or postoperative follow-up data for wound assessment. The detailed distribution of excluded patients according to the exclusion criteria is presented in the Section 3 (Figure 1).

Of the 130 patients who underwent surgical treatment during the study period, 72 patients were excluded according to these predefined criteria, resulting in a final cohort of 58 patients. Despite the notable proportion of excluded patients, these meticulous exclusion criteria were deliberately implemented to reduce the substantial sources of clinical and biological heterogeneity and to minimize potential confounding by factors known or likely to substantially influence postoperative wound healing and the systemic inflammatory response. Consequently, the final cohort was designed to represent a more clinically defined population, thereby enabling the examination of associations between host-related biological markers and postoperative wound complications.

### 2.2. Surgical Management and Study Standardization

The indication for operative treatment was established according to widely accepted principles described in the trauma literature [2,3,12,13,14,15]. To optimize the condition of the soft tissues before definitive fixation, all patients were initially managed with a short-leg splint, limb elevation, and regular soft-tissue assessment. The exact interval between injury and surgery was not available for all patients; however, definitive surgery was routinely deferred until soft-tissue swelling had adequately subsided and the wrinkle sign indicated that the soft tissues were suitable for surgical intervention.

To minimize treatment-related variability, all procedures were performed at the same institution, a tertiary trauma center, during an 11-month period within the same calendar year by the same orthopedic trauma team under the supervision of the same senior orthopedic trauma surgeon. Whenever appropriate according to fracture configuration, fixation was performed using a single implant system from the same manufacturer throughout the study period. All procedures were performed under pneumatic tourniquet control, and the tourniquet time did not exceed two hours in any patient. No patient received perioperative blood transfusion. Standardized perioperative protocols, including anesthesia, antibiotic prophylaxis, surgical preparation, and postoperative inpatient care, were consistently applied to all patients. Perioperative fluid management was routinely planned and administered by the anesthesia team according to standard clinical practice; however, detailed data regarding the type and volume of perioperative fluid administration were not available for retrospective analysis.

Postoperatively, all patients were immobilized in a short-leg splint until satisfactory soft-tissue healing had been achieved, which routinely occurred approximately three weeks after surgery. Sutures were then removed and supervised ankle range-of-motion exercises were initiated. Progressive weight-bearing and strengthening exercises were subsequently introduced in a controlled manner between the sixth and eighth postoperative weeks according to the patient’s clinical and radiographic progress.

All patients remained hospitalized until at least three consecutive wound inspections and dressing changes confirmed an uncomplicated early postoperative course. Patients without evidence of wound-related complications were subsequently discharged according to the institutional rehabilitation protocol. Hospitalization was prolonged whenever wound-related or other postoperative complications required additional treatment or closer clinical observation.

Routine outpatient follow-up was scheduled at 3 weeks, 6 weeks, 3 months, 6 months, and 1 year after surgery. At each visit, wound healing was evaluated clinically, and fracture healing was assessed radiographically. Additional investigations and appropriate interventions were undertaken whenever wound-related complications, delayed fracture healing, or other postoperative complications were suspected.

As the primary objective of this study was to investigate the contribution of host-related biological factors to postoperative wound healing, substantial efforts were made to minimize variability arising from surgical and perioperative factors. Standardizing the timing of surgery, operative technique, implant selection, postoperative rehabilitation, and follow-up protocol reduced potential procedural confounding and allowed a more reliable assessment of the relationship between routinely available hematological biomarkers and postoperative wound complications. Collectively, these standardization measures were intended to enhance the internal validity of this study by reducing variability related to surgical management and allowing a more focused assessment of associations between routinely available hematological biomarkers and postoperative wound complications.

### 2.3. Outcome Definition

The primary outcome of interest was the occurrence of postoperative wound complications (WoC). As the objective of the present study was to investigate host-related factors associated with impaired early postoperative wound healing rather than to compare individual wound complication types, clinically relevant wound-related manifestations were evaluated collectively as a composite outcome.

Postoperative wound complications were defined as wound dehiscence, prolonged serous wound drainage requiring continued wound dressing changes for more than seven consecutive days, clinically diagnosed superficial wound infection requiring systemic antibiotic therapy, deep wound infection requiring surgical debridement and/or implant removal, or clinically diagnosed osteomyelitis requiring medical and/or surgical treatment. In contrast, transient serous wound drainage that resolved spontaneously within the first seven postoperative days was considered part of the normal physiological healing response and was therefore not classified as a wound complication.

The individual wound manifestations were not considered mutually exclusive or independent events. In some patients, wound-related problems represented the sequential stages of the same clinical course; for example, prolonged serous drainage could be followed by wound dehiscence and subsequently by superficial or deeper infection. Therefore, these manifestations were not analyzed as separate events. The occurrence of any predefined wound-related complication during the primary observation period classified the patient as having WoC, and each patient was counted only once in the composite outcome. This approach was adopted to avoid artificially inflating the number of events and to preserve the patient as the unit of analysis, particularly given the limited number of patients with WoC (*n* = 18). The composite endpoint was intended to capture the clinically meaningful impairment of early wound healing and did not imply equivalence in severity or pathophysiology among its individual manifestations.

The treating orthopedic team assessed wound complications during hospitalization and routine outpatient follow-up. The diagnosis of individual wound manifestations was based on the clinical appearance and course of the wound and the treatment required, rather than on a separate research-specific adjudication system. Prolonged serous drainage was defined as wound drainage persisting for more than seven consecutive days and requiring continued dressing changes. When the drainage became purulent or foul-smelling and was considered clinically consistent with a superficial wound infection, systemic antibiotic treatment was initiated. Persistent or progressive wound drainage accompanied by clinical findings suggestive of deeper involvement was considered a deep wound infection when surgical debridement and/or implant removal was required. Osteomyelitis was diagnosed clinically by the treating team and managed with medical and/or surgical treatment. As these wound manifestations could evolve sequentially within the same clinical course, they were not considered mutually exclusive or independent events and were not separately analyzed.

The primary observation window was defined as the first three postoperative weeks, corresponding to the early postoperative wound-healing period during which patients were under close clinical surveillance and routine wound care, including assessment until suture removal. Wound-related events occurring after this predefined period were not included in the primary outcome analysis. Although patients continued to receive routine outpatient follow-up thereafter, the relationship of such later events to the initial postoperative wound-healing process could not be reliably established, as they may be affected by subsequent patient-, treatment-, trauma-, or environmental-related factors after the early postoperative period. Moreover, later wound-related or infectious events were not systematically recorded as a separate outcome in the original retrospective dataset and therefore could not be reliably quantified. Accordingly, the present study was designed to evaluate early postoperative wound complications rather than the cumulative incidence of wound-related complications throughout the entire follow-up period.

Complications unrelated to wound healing, including delayed union, nonunion, malunion, fixation failure, implant failure, loss of reduction, complex regional pain syndrome, postoperative joint stiffness, suboptimal functional recovery, thromboembolic events, and other non-wound-related postoperative complications, were intentionally excluded because they primarily reflect biomechanical, neurological, or rehabilitation-related mechanisms rather than alterations in host biology relevant to wound repair. Accordingly, the outcome definition was deliberately restricted to complications directly reflecting impaired soft-tissue healing, allowing a more specific evaluation of associations between host-related biological factors and wound healing while minimizing the influence of unrelated postoperative events.

### 2.4. Clinical Variables and Perioperative Laboratory Assessment

Demographic variables, including age and sex, were recorded for all patients. Routine perioperative laboratory data were retrieved from the institutional electronic medical record system. Blood samples obtained within the final 24 h before surgery were considered to represent each patient’s baseline biological status, whereas blood samples routinely collected within the first six postoperative hours were considered to reflect the early systemic biological response to surgical injury. All laboratory analyses were performed as part of routine clinical care using standardized institutional laboratory protocols. Complete blood count parameters were measured using the Siemens ADVIA 2120 hematology analyzer (Siemens Healthineers, Erlangen, Germany), whereas clinical chemistry parameters were analyzed using the Siemens Atellica CH system (Siemens Healthineers, Erlangen, Germany). In instances where multiple measurements were obtained within the predefined preoperative or postoperative sampling window, the initial result available was selected for analysis. If the initial sample was reported as clotted and a repeat measurement was subsequently performed as part of routine clinical care, the repeat result was used.

The evaluated hematological parameters included white blood cell count (3.9–10.2 × 10^9^/L), absolute neutrophil count (1.5–7.7 × 10^9^/L), absolute lymphocyte count (1.1–4.5 × 10^9^/L), absolute monocyte count (0.1–0.9 × 10^9^/L), absolute eosinophil count (0.02–0.5 × 10^9^/L), absolute basophil count (0–0.2 × 10^9^/L), hemoglobin (13.5–17.2 g/dL for males; 12.0–15.6 g/dL for females), hematocrit (39.5–50.5% for males; 35.5–45.5% for females), platelet count (150–400 × 10^9^/L), and delta neutrophil index (DNI). The evaluated biochemical parameters included sodium (132–146 mEq/L), potassium (3.5–5.5 mEq/L), calcium (8.7–10.4 mg/dL), chloride (99–109 mEq/L), urea (19–49 mg/dL), creatinine (0.7–1.3 mg/dL for males; 0.5–1.1 mg/dL for females), total protein (57–82 g/L), and albumin (32–48 g/L).

In addition to individual laboratory parameters, several composite biomarkers were calculated because they integrate different aspects of the host biological response. Neutrophil-to-lymphocyte ratio (NLR) and the systemic immune-inflammation index (SII) were evaluated as markers of systemic inflammatory and immune activation. NLR was calculated as the absolute neutrophil count (×10^9^/L) divided by the absolute lymphocyte count (×10^9^/L), whereas the SII was calculated as platelet count (×10^9^/L) × absolute neutrophil count (×10^9^/L)/absolute lymphocyte count (×10^9^/L). The prognostic nutritional index (PNI) and the hemoglobin, albumin, lymphocyte, and platelet (HALP) score were evaluated as indicators of nutritional and immunological status. The PNI was calculated as serum albumin (g/L) + 5 × absolute lymphocyte count (×10^9^/L), whereas the HALP score was calculated as hemoglobin (g/dL) × albumin (g/L) × absolute lymphocyte count (×10^9^/L)/platelet count (×10^9^/L). Each composite biomarker was calculated using both preoperative and early postoperative laboratory measurements, allowing the separate assessment of the patient’s baseline biological condition and the acute physiological response to surgical injury.

For all analyses, laboratory parameters and composite biomarkers were evaluated independently according to sampling time (preoperative versus early postoperative) and subsequently compared between patients who developed postoperative wound complications and those who did not.

### 2.5. Statistical Analysis

Statistical analyses were performed using IBM SPSS Statistics for Windows (Version 26.0; IBM Corp., Armonk, NY, USA). The distribution of continuous variables was assessed using the Kolmogorov–Smirnov test together with visual inspection of histograms. Owing to the skewed distribution of continuous variables, non-parametric statistical methods were used throughout the analyses. Continuous variables are presented as medians (interquartile ranges [IQRs]) and minimum–maximum ranges, whereas categorical variables are presented as frequencies and percentages. Comparisons between patients with and without postoperative WoC were performed using the Mann–Whitney U test for continuous variables and Pearson’s chi-square test or Fisher’s exact test for categorical variables, as appropriate.

No biomarker was prespecified as a primary hypothesis; all biomarker analyses were exploratory. To account for the potential impact of multiple testing, a Benjamini–Hochberg false-discovery-rate (FDR) adjustment was applied to the univariate biomarker comparisons. FDR adjustment was performed separately for the preoperative and early postoperative biomarker sets. FDR-adjusted *p* values were reported alongside the original *p* values and were considered in the selection of candidate variables for multivariable and ROC analyses. ROC curve analyses were subsequently performed only for biomarkers that remained statistically significant after FDR adjustment. The area under the ROC curve (AUC), 95% confidence intervals (95% CIs), optimal cutoff values determined according to the Youden index, sensitivity, and specificity were calculated.

Two separate multivariable models were constructed to evaluate preoperative and early postoperative factors associated with postoperative wound complications. Candidate biomarkers were selected based on nominal univariate significance and retention of statistical significance after FDR adjustment. Clinically relevant demographic variables, such as sex, were considered separately from the FDR-based biomarker selection criterion. Before model construction, variables representing overlapping biological information were reviewed to minimize multicollinearity. Accordingly, variables reflecting analogous biological processes, such as sodium versus chloride, and individual inflammatory cell counts versus composite inflammatory indices, were not entered simultaneously. In the early postoperative model, NLR was retained as the composite inflammatory marker, and additional inflammatory cell parameters with overlapping biological information were not entered simultaneously. Given the limited number of outcome events and the potential for small-sample bias and separation with conventional logistic regression, Firth penalized logistic regression was used for both multivariable models. The analyses were performed in R using the logistf package. Firth regression coefficients (Bs), standard errors (SEs), odds ratios (ORs), 95% CIs, and *p* values were reported. Given the limited number of outcome events and exploratory study design, the multivariable analyses were considered exploratory and were intended to identify potential independent associations rather than to develop a definitive clinical prediction model. A two-sided *p* value of <0.05 was considered statistically significant for the primary univariate and multivariable analyses, whereas an FDR-adjusted *p* value of <0.05 was considered statistically significant for the FDR-adjusted analyses.

During the preparation of this manuscript, ChatGPT (GPT-5.2; OpenAI, San Francisco, CA, USA) was used exclusively for minor language editing limited to spelling, grammar, and punctuation and was not used for the conception, design, execution, analysis, interpretation, drafting, or formulation of the study.

## 3. Results

The detailed inclusion and exclusion process of the study population is presented in Figure 1. Of the 58 patients included in the final cohort, 12 (20.7%) had isolated malleolar fractures, 28 (48.3%) bimalleolar fractures, 3 (5.2%) trimalleolar fractures, 5 (8.6%) distal tibial metaphyseal fractures, 4 (6.9%) talar fractures, and 6 (10.3%) calcaneal fractures.

Among the 58 patients, postoperative WoC developed in 18 (31.0%) patients, whereas uncomplicated wound healing was observed in 40 (69.0%). Univariate analysis demonstrated that patients who developed postoperative WoC had significantly lower preoperative hemoglobin, hematocrit, sodium, and chloride levels, whereas preoperative platelet counts and total protein concentrations were significantly higher than those in those without WoC (all *p* < 0.05). Conventional biochemical hyponatremia, defined as a serum sodium level of <135 mEq/L, was uncommon in the cohort; four patients (6.9%) had preoperative hyponatremia. No significant differences were observed regarding age, white blood cell count, leukocyte subpopulations, inflammatory markers assessed in this study, or composite inflammatory indices. In addition, female sex was significantly more frequent among patients who developed postoperative WoC (*p* < 0.001). After Benjamini–Hochberg FDR adjustment of the preoperative biomarker comparisons, only platelet count and total protein remained statistically significant, whereas the other nominally significant biomarker associations did not (Table 1).

During the early postoperative period, patients who developed WoC exhibited significantly lower hemoglobin, hematocrit, lymphocyte, basophil, sodium, chloride, creatinine, PNI, and HALP values, whereas their NLR was significantly higher (all *p* < 0.05). Only two patients (3.4%) had early postoperative serum sodium levels below 135 mEq/L, indicating that conventional biochemical hyponatremia was uncommon during this period. After Benjamini–Hochberg FDR adjustment of the early postoperative biomarker comparisons, sodium, absolute basophil count, chloride, hemoglobin, and NLR remained statistically significant, whereas the associations observed for hematocrit, PNI, HALP, creatinine, and absolute lymphocyte count did not retain statistical significance (Table 2).

ROC curve analysis demonstrated that, among the preoperative biomarkers, total protein showed the highest discriminatory performance for postoperative wound complications (AUC = 0.886), followed by platelet count (AUC = 0.767). Among the early postoperative biomarkers, sodium demonstrated the greatest discriminatory ability (AUC = 0.808), followed by absolute basophil count (AUC = 0.733), chloride (AUC = 0.722), hemoglobin (AUC = 0.711), and NLR (AUC = 0.711). The optimal cutoff values together with their corresponding sensitivity and specificity are presented in Table 3.

Among the variables that reached statistical significance in the univariate analyses and remained significant after FDR adjustment, biomarkers reflecting overlapping biological information were reviewed for potential multicollinearity before multivariable analysis. Consequently, platelet count and total protein were retained for the preoperative multivariable model, while variables that did not retain statistical significance after FDR adjustment were excluded. In the preoperative Firth model, female sex (OR = 7.209, 95% CI 1.189–57.415; *p* = 0.031) and higher preoperative total protein levels (OR = 1.399, 95% CI 1.168–1.788; *p* < 0.001) were associated with postoperative WoC. Preoperative platelet count was not statistically significant in the Firth model (Table 4).

In the early postoperative Firth model, hemoglobin, NLR, and sodium were included in the multivariable analysis. Although absolute basophil count remained statistically significant after FDR adjustment, it was not included because NLR was retained as the composite inflammatory marker and additional inflammatory parameters with overlapping biological information were not entered simultaneously. Lower serum sodium (OR = 0.477, 95% CI 0.241–0.789; *p* = 0.001) was associated with postoperative WoC. In contrast, hemoglobin and NLR were not significantly associated with the outcome in the Firth model (Table 5).

## 4. Discussion

Postoperative wound complications remain a clinically important challenge across surgical disciplines and are influenced by a complex interplay of local tissue conditions, procedural factors, and patient-related characteristics. The present study was designed to investigate the contribution of host-related biological factors to postoperative wound healing while minimizing variability arising from anatomical location, surgical technique, and perioperative management. By using the peri-ankle region as a clinically relevant model with a well-recognized susceptibility to wound complications [2,3,11,12,13,14], we sought to examine associations between patient-related biological factors and wound healing within a clinically restricted surgical cohort. The principal findings of the present study were twofold. First, female sex and higher preoperative total protein levels were associated with the development of postoperative WoC in the preoperative Firth model. Second, lower serum sodium in the early postoperative period was associated with WoC in the early postoperative Firth model.

Among the evaluated preoperative laboratory parameters, univariate analyses identified significant associations between WoC and serum hemoglobin, hematocrit, platelet count, sodium, chloride, and total protein levels. After Benjamini–Hochberg FDR adjustment, only preoperative platelet count and total protein remained statistically significant, whereas the associations observed for hemoglobin, hematocrit, sodium, and chloride did not retain statistical significance after FDR adjustment. However, after multivariable adjustment using Firth penalized logistic regression, higher preoperative total protein (OR = 1.399, 95% CI 1.168–1.788; *p* < 0.001) remained associated with postoperative WoC. Preoperative total protein also showed the best discriminatory performance among all baseline preoperative parameters (AUC = 0.886), with an optimal cutoff value of ≥68.5 g/L, corresponding to a sensitivity of 88.9% and a specificity of 85%. Because these ROC analyses and optimal cutoff values were both derived and evaluated within the same cohort, they should be considered exploratory and hypothesis-generating rather than validated clinical decision thresholds. Accordingly, these findings require confirmation and external validation in larger prospective cohorts before they can be recommended for clinical risk stratification or routine clinical use. To our knowledge, few studies have examined preoperative total protein in relation to postoperative wound or infectious complications. However, the direction of the association observed in our study differs from that reported in the previous literature. In 2017, Munteanu et al. reported that lower preoperative total protein and albumin levels were associated with surgical wound infection and overall postoperative complications in patients with gastric cancer, while postoperative total protein and albumin levels were associated with mortality [16]. Similarly, in 2016, Nakagawa et al. reported that lower postoperative total protein levels measured on postoperative day 7 and lower postoperative albumin levels measured on postoperative days 14 and 21 were associated with the development of surgical site infections following colorectal surgery [17]. Thus, unlike these previous findings, our study identified higher preoperative total protein levels as being associated with postoperative WoC. Several previous investigations have largely focused on serum albumin and other nutritional markers. In a 2019 meta-analysis, Gu et al. reported that malnutrition and hypoalbuminemia were associated with an increased risk of postoperative WoC [18]. He et al. also reported that preoperative and postoperative albumin levels were associated with the development of surgical wound dehiscence following spinal surgery [19]. In a meta-analysis published in 2017, Yuwen et al. analyzed outcomes from 112,183 patients and reported that hypoalbuminemia was an important risk factor for surgical site infection [20]. Interestingly, albumin was not associated with WoC in our cohort, whereas total protein remained independently associated with the outcome. A partially different pattern was reported by Huda et al., who investigated surgical site infections in 112 patients undergoing elective laparotomy and found an association between preoperative serum total protein levels and surgical site infection, whereas preoperative albumin levels were not significantly associated with the outcome [21]. However, the biological significance of this finding in the present cohort remains uncertain. While albumin primarily reflects nutritional status and, to some extent, systemic inflammation, total protein also incorporates circulating globulin fractions, including immunoglobulins, complement proteins, and acute-phase reactants, and therefore does not represent a specific nutritional or inflammatory biomarker. The unexpected direction of the association in our study, with higher rather than lower total protein levels associated with WoC, further limits a straightforward nutritional interpretation. Although differences in globulin fractions or inflammatory proteins could theoretically contribute to this observation, these mechanisms cannot be evaluated in the present dataset because CRP, serum protein fraction, and electrophoresis data were not available. The association may alternatively reflect residual confounding, unmeasured clinical characteristics, perioperative measurement context, or chance. Therefore, the association between higher preoperative total protein and WoC should be regarded as an unexpected exploratory finding rather than evidence that total protein independently reflects a specific biological pathway or constitutes a clinically actionable nutritional biomarker. Further studies incorporating serum protein electrophoresis together with more comprehensive inflammatory and immunological profiling are warranted to clarify the underlying biological mechanisms.

Another noteworthy finding of the present study was the significant association between female sex and postoperative WoC in the univariate analysis. Furthermore, after multivariable adjustment using Firth penalized logistic regression, female sex remained statistically significantly associated with postoperative WoC (unadjusted OR = 10.500, 95% CI 2.800–39.374; *p* < 0.001; adjusted OR = 7.209, 95% CI 1.189–57.415; *p* = 0.031). Although the adjusted effect estimate remained large, the relatively wide confidence interval and the limited number of WoC events, particularly the small number of events among male patients, warrant cautious interpretation and suggest potential small-sample or sparse-data-related instability. Although sex has been investigated as a potential risk factor following ankle fracture surgery [22,23,24], previous studies have reported inconsistent findings, and other patient- and injury-related factors, including obesity, comorbidity burden, and age, have more consistently been associated with postoperative wound complications [25,26,27]. In 2019, Aghdassi et al. analyzed data from 1,266,782 individuals and reported male sex as a risk factor for surgical site infection following trauma and abdominal surgery, whereas female sex was identified as a risk factor in cardiovascular surgery [28]. Therefore, the association between female sex and postoperative WoC observed in our cohort should be interpreted cautiously and should not be considered evidence of a definitive independent biological effect. Several biological and functional factors may theoretically contribute to sex-related differences in wound healing; however, these mechanisms could not be evaluated in the present dataset. In particular, menopausal status, body composition, lower-extremity muscle mass, microvascular characteristics, and postoperative activity or adherence were not available for analysis, precluding the assessment of potential explanations for the observed sex-related association. Accordingly, female sex should be regarded as an exploratory association requiring external validation rather than definitive evidence of an independent biological risk factor for postoperative WoC. Further studies with larger prospective cohorts and comprehensive assessment of body composition, menopausal status, comorbidity burden, and postoperative functional factors are required to determine whether this association is reproducible and clinically meaningful.

Serum sodium was not retained in the preoperative Firth multivariable model because its FDR-adjusted *p* value did not remain statistically significant. In contrast, serum sodium remained FDR-significant in the early postoperative analysis and was also associated with postoperative WoC in the early postoperative Firth model, demonstrating moderate discriminatory performance (AUC = 0.808). A postoperative sodium cutoff value of ≤138 mEq/L yielded a sensitivity of 77.8% and a specificity of 70.0%. Importantly, this data-derived cutoff should not be interpreted as a conventional clinical definition of hyponatremia or as a clinically actionable threshold. Moreover, these findings should be interpreted cautiously because serum sodium is a dynamic perioperative laboratory parameter and may be influenced by several treatment- and patient-related factors, including intravenous fluid administration, perioperative fasting, blood loss, medications, renal function, and stress-related physiological changes. Detailed information regarding the type and volume of perioperative fluid administration, quantitative blood loss, and several potentially relevant clinical factors was not available for retrospective analysis. Therefore, the observed association between lower serum sodium and WoC cannot be interpreted as evidence of an intrinsic host biological vulnerability or as a direct causal relationship. On the other hand, in contrast to total protein and female sex, our findings regarding serum sodium are more consistent with the existing literature. Preoperative hyponatremia has repeatedly been associated with an increased risk of postoperative complications across multiple surgical specialties [26,29,30,31,32]. However, most previous studies have focused on overall morbidity or mortality rather than wound-specific outcomes [33,34,35], and the present findings should not be interpreted as establishing a similar causal relationship for wound healing. Our findings extend these observations by demonstrating that early postoperative serum sodium was associated with postoperative WoC and showed moderate discriminatory performance in our cohort. The basis of this association is likely multifactorial, and the association between lower sodium levels and WoC warrants further investigation, but its biological basis remains uncertain and may reflect underlying patient characteristics, perioperative management, or other unmeasured confounding factors. Prospective studies with standardized perioperative fluid management and systematic assessment of renal function, medications, pre-existing electrolyte abnormalities, and other relevant clinical variables are required to determine whether serum sodium provides independent information regarding early postoperative wound complications.

From a clinical perspective, the findings of the present study should be regarded as hypothesis-generating rather than as evidence supporting immediate clinical implementation. Although the biomarkers evaluated in this study are routinely available as part of standard perioperative care and therefore could be readily incorporated into clinical assessment, the present findings do not establish their clinical utility or justify biomarker-based modification of perioperative management. In particular, the data-derived ROC thresholds were derived and evaluated within the same small cohort and should not be interpreted as clinically actionable thresholds. The observed associations of female sex and preoperative total protein with postoperative WoC, as well as the association of early postoperative serum sodium with WoC, should therefore be considered exploratory findings requiring confirmation in larger prospective cohorts. Prospective studies with larger sample sizes, standardized perioperative management, comprehensive assessment of established clinical risk factors, and external validation are required before these variables can be considered for clinical risk stratification. Future research should also determine whether incorporating these biomarkers and demographic factors into established clinical risk models provides incremental predictive value beyond routinely recognized patient- and treatment-related risk factors.

Several limitations of the present study should be acknowledged. First, the retrospective single-center design and relatively small sample size limit the generalizability of the findings. The limited number of WoC events may have reduced statistical power and the precision of the estimated odds ratios, particularly for variables demonstrating relatively large effect sizes, and may have increased the risk of sparse-data-related instability despite the use of Firth penalized logistic regression to reduce small-sample bias and separation. Consequently, the multivariable analyses should be interpreted as exploratory analyses intended to identify potential host-related biological factors rather than to establish a definitive clinical prediction model. Validation in larger prospective cohorts will be necessary before these findings can be translated into routine clinical practice. Second, postoperative wound complications are inherently multifactorial and may be influenced by a broad range of patient-related, biological, injury-related, and treatment-related confounders. We therefore deliberately prioritized the establishment of a clinically restricted study cohort with predefined eligibility criteria, even at the cost of reducing the sample size, by excluding major clinical factors that could substantially alter wound-healing biology, including poorly controlled diabetes mellitus, open fractures, symptomatic peripheral vascular disease, chronic inflammatory or autoimmune disorders, immunosuppressive therapy, and patients requiring multiple surgical approaches. However, the application of relatively strict exclusion criteria led to the exclusion of over half of the initially identified surgically treated patients. While this approach reduced major sources of clinical and biological heterogeneity, it also substantially narrowed the study population and may limit the applicability of our findings to more heterogeneous trauma populations. Nevertheless, not all potential biological confounders could be identified or adequately controlled for, and several potentially relevant patient-related and perioperative factors were not available in sufficient detail for analysis, including smoking status, body mass index, ASA classification, controlled diabetes status, exact time from injury to surgery, operative duration, quantitative blood loss, exact tourniquet duration, and perioperative fluid administration. The absence of detailed information on several established wound-healing risk factors therefore leaves the possibility of residual confounding. Third, although anatomically related peri-ankle fractures were analyzed as a single cohort to improve statistical power, individual fracture types may differ in terms of soft-tissue injury, vascularity, and wound-healing characteristics. Several individual fracture categories contained very few patients, which precluded statistically reliable subgroup analyses according to fracture diagnosis and made inclusion of these categories as separate covariates in the multivariable models inappropriate given the limited number of outcome events. Thus, potential differences among individual peri-ankle fracture types could not be explored. This strategy represents a trade-off between internal validity and external generalizability.

Fourth, only routinely available laboratory biomarkers were evaluated; more specific inflammatory and immunological biomarkers, such as C-reactive protein, cytokines, and serum protein electrophoresis, were unavailable and therefore could not be incorporated into the analyses. Furthermore, although both preoperative and early postoperative laboratory biomarkers were evaluated, perioperative changes were not analyzed. The influence of these changes is multifactorial, being shaped not only by the host biological response but also by intraoperative and postoperative fluid management, intraoperative blood loss, and blood transfusion. In particular, several factors that may influence perioperative serum sodium levels were not available in sufficient detail for analysis, including the type and volume of perioperative intravenous fluids, quantitative blood loss, pre-existing electrolyte disturbances, relevant medication use, and detailed renal or comorbidity profiles. Consequently, the association between early postoperative serum sodium and WoC cannot be separated from potential treatment-related or underlying clinical factors. Similarly, the unexpected association between higher total protein and WoC should be interpreted cautiously because specific protein fractions and inflammatory markers such as CRP were unavailable. Subsequent studies assessing dynamic perioperative biomarker alterations may offer further insight into the relationship between the physiological response to surgical stress and postoperative wound complications. Finally, although patients continued to receive routine outpatient follow-up beyond the primary three-week observation period, later wound-related or infectious events were not systematically recorded as a separate outcome in the retrospective dataset. Therefore, the present study cannot reliably estimate the cumulative incidence of wound complications throughout the entire follow-up period or determine whether later events were related to the initial postoperative wound-healing response.

Despite these limitations, the present study also exhibits several significant strengths. To the best of our knowledge, this is one of the few studies to simultaneously evaluate both preoperative biological status and the early postoperative biological response using routinely available laboratory biomarkers in a clinically restricted fracture cohort with predefined eligibility criteria. Additional strengths include standardized surgical management, the establishment of a clearly defined wound-related outcome framework, and the evaluation of inexpensive, readily available biomarkers that could be readily incorporated into routine clinical assessment. In order to reduce major sources of clinical and biological heterogeneity, the eligibility criteria were deliberately defined to exclude patients with conditions that could substantially influence wound healing or the perioperative inflammatory response. This resulted in the exclusion of 72 of the 130 patients initially screened (55.4%). It is acknowledged that this extensive restriction may introduce selection bias and limit the external validity and direct applicability of the findings to the broader population of surgically treated peri-ankle fractures. Nevertheless, this approach was deemed essential to establish a more clinically defined study cohort, in which associations between routinely available host-related biomarkers and postoperative wound complications could be examined with fewer major competing sources of variation. Of particular note is the manner in which all excluded patients and the specific reasons for exclusion are presented in the study flowchart (Figure 1), thus allowing the extent and nature of this selection process to be clearly assessed. Furthermore, the minimization of variability pertaining to anatomical location, surgical technique, and postoperative management enabled a more focused evaluation of the associations between host-related biological factors and postoperative wound healing.

## 5. Conclusions

In this study’s clinically restricted cohort of patients undergoing peri-ankle fracture surgery, female sex and higher preoperative serum total protein levels were associated with postoperative wound complications, while lower early postoperative serum sodium was also associated with the outcome. These findings should be considered exploratory and do not establish causal or intrinsic host biological mechanisms underlying wound complications. The observed associations, particularly the data-derived biomarker thresholds, require confirmation in larger prospective cohorts with standardized perioperative assessment and external validation before their potential clinical utility can be determined.

## Figures and Tables

**Table 1:** Baseline clinical characteristics and preoperative laboratory biomarkers according to postoperative wound complications.

Variable		No Wound Complications (*n* = 40)	Wound Complications (*n* = 18)	*p*	FDR-Adjusted *p*
Age (years)		48.5 (22) (28–71)	51 (11) (24–71)	0.711	N/A
Sex	Female	10 (25%)	14 (77.8%)	<0.001	N/A
	Male	30 (75%)	4 (22.2%)		
Hemoglobin (g/dL)		14.55 (1.4) (11.2–15.7)	13.4 (2.4) (10.7–17.5)	0.037	0.136
Hematocrit (%)		43.55 (3.9) (34.9–47.4)	41.8 (6.95) (32–51.3)	0.029	0.128
Platelet Count (×10^9^/L)		242.5 (107) (155–399)	276 (118.25) (255–434)	0.001	0.011
White Blood Cell Count (×10^9^/L)		11.13 (3.52) (6.2–16.09)	9.83 (4.54) (6.19–16.24)	0.400	0.600
Neutrophil Count (×10^9^/L)		8.14 (4.99) (3.35–12.2)	8.11 (2.6) (4.46–10.73)	0.439	0.660
Lymphocyte Count (×10^9^/L)		1.96 (1.22) (0.92–4.61)	1.81 (1.88) (0.81–4.97)	0.920	0.946
Monocyte Count (×10^9^/L)		0.54 (0.25) (0.33–1.07)	0.5 (0.29) (0.28–0.75)	0.480	0.660
Eosinophil Count (×10^9^/L)		0.09 (0.14) (0.02–0.23)	0.04 (0.12) (0.02–0.2)	0.188	0.460
Basophil Count (×10^9^/L)		0.03 (0.01) (0.01–0.06)	0.02 (0.02) (0–0.08)	0.068	0.187
Sodium (mEq/L)		140 (3) (136–143)	138 (4.75) (134–141)	0.015	0.105
Potassium (mEq/L)		4.25 (0.5) (3.7–4.7)	4.2 (0.42) (3.8–4.8)	0.946	0.946
Calcium (mg/dL)		9.1 (0.4) (8.4–9.9)	9.3 (0.38) (8.7–9.9)	0.051	0.160
Chloride (mEq/L)		106.5 (4) (102–113)	105 (5.5) (98–108)	0.019	0.105
Urea (mg/dL)		34 (13) (18–55)	28 (15.75) (21–56)	0.223	0.491
Creatinine (mg/dL)		0.8 (0.22) (0.46–1.12)	0.77 (0.26) (0.46–1.33)	0.762	0.838
Total Protein (g/L)		65.5 (4.75) (61–76)	72 (3.5) (66–78)	<0.001	0.011
Albumin (g/L)		43 (3) (39–47)	44 (3.25) (39–47)	0.435	0.660
NLR		3.74 (3.1) (1.5–12.84)	4.17 (1.58) (2.03–10.16)	0.737	0.838
DNI (%)		0 (0.9) (0–5.3)	0 (1.63) (0–6.6)	0.712	0.838
SII		1020.68 (1067.05) (269.43–2196)	1236.66 (848.27) (839.66–2662.05)	0.459	0.660
PNI		52.80 (6.95) (43.6–66.05)	54.05 (9.24) (44.35–68.85)	0.726	0.838
HALP		4.5189 (2.83) (2.31–9.74)	4.2387 (3.99) (1.08–9)	0.420	0.660

Data are presented as median (IQR) (minimum–maximum) for continuous variables and as *n* (%) for categorical variables. *p*: statistical significance value; FDR-adjusted *p*: Benjamini–Hochberg false-discovery-rate-adjusted *p* value; N/A: not applicable; *n*: number of patients. A two-sided *p* < 0.05 was considered statistically significant for the original univariate analyses; FDR-adjusted *p* < 0.05 was considered statistically significant in the FDR analysis.

**Table 2:** Early postoperative laboratory biomarkers according to postoperative wound complications.

Variable	No Wound Complications (*n* = 40)	Wound Complications (*n* = 18)	*p*	FDR-Adjusted *p*
Hemoglobin (g/dL)	12.7 (2.9) (10–15.3)	11.3 (2.1) (10.3–13.6)	0.011	0.048
Hematocrit (%)	38.6 (8.5) (29.9–48.9)	35.2 (4.07) (32.5–40)	0.014	0.051
Platelet Count (×10^9^/L)	256 (184) (143–558)	255 (111.5) (217–714)	0.638	0.668
White Blood Cell Count (×10^9^/L)	10.64 (7.21) (4.21–18.91)	11.71 (2.9) (8.72–20.52)	0.122	0.168
Neutrophil Count (×10^9^/L)	7.76 (6.31) (2.51–15.64)	9.76 (2.5) (6.24–18.06)	0.106	0.155
Lymphocyte Count (×10^9^/L)	1.76 (1.12) (0.61–3.38)	1.09 (0.48) (0.83–1.8)	0.047	0.103
Monocyte Count (×10^9^/L)	0.6 (0.23) (0.29–0.99)	0.81 (0.61) (0.23–1.23)	0.439	0.537
Eosinophil Count (×10^9^/L)	0.08 (0.07) (0.01–0.48)	0.04 (0.08) (0.01–0.27)	0.053	0.106
Basophil Count (×10^9^/L)	0.02 (0.03) (0.01–0.06)	0.01 (0.01) (0–0.02)	0.003	0.029
Sodium (mEq/L)	140 (3) (137–144)	138 (1.75) (132–140)	<0.001	0.022
Potassium (mEq/L)	4.3 (0.5) (3.6–5.2)	4.1 (0.035) (3.7–4.8)	0.398	0.515
Calcium (mg/dL)	8.6 (0.7) (7.9–9.2)	8.4 (0.3) (7.8–9)	0.063	0.116
Chloride (mEq/L)	106 (3.75) (102–111)	103 (6.5) (100–108)	0.004	0.029
Urea (mg/dL)	32 (11) (15–41)	24 (16.5) (17–56)	0.490	0.548
Creatinine (mg/dL)	0.75 (0.32) (0.43–0.96)	0.63 (0.24) (0.48–1.12)	0.030	0.083
Total Protein (g/L)	61 (8) (51–69)	60 (13) (31–66)	0.761	0.761
Albumin (g/L)	39 (5) (30–45)	37 (5.75) (27–40)	0.498	0.548
NLR	4.5 (4.64) (1.17–17.51)	9.43 (4.26) (3.63–17.04)	0.011	0.048
DNI (%)	0 (0) (0–6.5)	0 (1.63) (0–6.3)	0.087	0.137
SII	1231.67 (2127.7) (191.66–4634.93)	2543.13 (2014.86) (874.33–12,164.94)	0.069	0.117
PNI	45.95 (8.45) (36.25–60.9)	42.4 (7.38) (35.6–49)	0.019	0.060
HALP	2.99 (2.96) (0.97–13.33)	2.18 (1.91) (0.6–3.35)	0.037	0.090

Data are presented as median (IQR) (minimum–maximum) for continuous variables and as *n* (%) for categorical variables. *p*: statistical significance value; FDR-adjusted *p*: Benjamini–Hochberg false-discovery-rate-adjusted *p* value; *n*: number of patients. A two-sided *p* < 0.05 was considered statistically significant for the original univariate analyses; FDR-adjusted *p* < 0.05 was considered statistically significant in the FDR analysis.

**Table 3:** Receiver operating characteristic analysis of perioperative laboratory biomarkers for the discrimination of postoperative wound complications.

Variable		Cutoff (Youden)	AUC	95% CI	Sensitivity (%)	Specificity (%)
Preoperative	Platelet	≥249 × 10^9^/L	0.767	0.647–0.886	100.0	55.0
	Total protein	≥68.5 g/L	0.886	0.797–0.976	88.9	85
Early Postoperative	Hemoglobin	≤13.15 g/dL	0.711	0.577–0.845	88.9	50.0
	Absolute basophil count	≤0.025 × 10^9^/L	0.733	0.606–0.861	100.0	40.0
	Sodium	≤138 mEq/L	0.808	0.694–0.923	77.8	70.0
	Chloride	≤102.5 mEq/L	0.722	0.572–0.873	44.4	95
	NLR	>4.98	0.711	0.577–0.845	88.9	55.0

AUC, area under the receiver operating characteristic curve; CI, confidence interval. AUC values are presented with 95% confidence intervals. Cutoff values were determined using the Youden index and should be considered exploratory, data-derived thresholds rather than clinically validated cutoffs. The basophil cutoff should be interpreted cautiously because values at this level may approach the analytical resolution of routine automated differential blood counts and may therefore be subject to measurement variability.

**Table 4:** Firth penalized logistic regression analysis of preoperative factors associated with postoperative wound complications.

Variable	Unadjusted OR (95% CI)	Firth B	Firth SE	Firth-Adjusted OR	95% CI	*p*
Female sex ^†^	10.500 (2.800–39.374)	1.975	0.978	7.209	1.189–57.415	0.031
Preoperative PLT	—	0.001	0.007	1.001	0.988–1.014	0.882
Preoperative total protein	—	0.336	0.105	1.399	1.168–1.788	<0.001

^†^ Reference category for sex: male. *p* < 0.05 indicates statistical significance. OR, odds ratio; CI, confidence interval;PLT, platelet count; SE, standard error. Multivariable estimates were obtained using Firth penalized logistic regression. The unadjusted OR for female sex was calculated from the corresponding 2 × 2 contingency table.

**Table 5:** Firth penalized logistic regression analysis of postoperative factors associated with postoperative wound complications.

Variable	Firth B	Firth SE	Firth-Adjusted OR	95% CI	*p*
Postoperative HGB	−0.297	0.238	0.743	0.457–1.159	0.193
Postoperative NLR	−0.009	0.092	0.991	0.825–1.181	0.917
Postoperative sodium	−0.741	0.289	0.477	0.241–0.789	0.001

OR, odds ratio; CI, confidence interval; HGB, hemoglobin; NLR, neutrophil-to-lymphocyte ratio; SE, standard error. Multivariable estimates were obtained using Firth penalized logistic regression.

## Data Availability

The datasets generated and/or analyzed during the current study are stored in a private repository and are not publicly available; however, they may be obtained from the corresponding author upon reasonable request.

## Supplementary Materials

The following supporting information can be downloaded at: https://www.mdpi.com/article/10.3390/jcm15197647/s1, Supplementary File S1: STROBE Statement—Checklist of items that should be included in reports of observational studies.

## Abbreviations

The following abbreviations are used in this manuscript:

WoC	Wound complications
ROC	Receiver operating characteristic
OR	Odds ratio
AUC	Area under the curve
STROBE	Strengthening the Reporting of Observational Studies in Epidemiology
ORIF	Open reduction and internal fixation
DNI	Delta neutrophil index
NLR	Neutrophil-to-lymphocyte ratio
SII	Systemic immune-inflammation index
PNI	Prognostic nutritional index
HALP	Hemoglobin, albumin, lymphocyte, and platelet score
IQR	Interquartile range
FDR	Benjamini–Hochberg false discovery rate
CI	Confidence interval
DM	Diabetes mellitus
DFI	Diabetic foot infection
PVD	Peripheral vascular disease
