# Supplementary material for: Associations of Preoperative and Early Postoperative Biomarkers with Wound Complications in a Clinically Restricted Surgical Population: A Retrospective Cohort Study
Source: J Clin Med. 2026 Oct 2;15(19):7647. doi: 10.3390/jcm15197647 (PMC13666511; doi:10.3390/jcm15197647)
Supplement: Supplementary file 1 [file jcm-15-07647-s001.zip › jcm-4523114-supplementary.pdf]

STROBE Statement—Checklist of items that should be included in reports of observational studies

Study: Host Biology and Postoperative Wound Complications: Insights from Inflammatory and Nutritional Biomarkers

Item No.	Recommendation	Page No.	Relevant text from manuscript
1a	Indicate the study's design with a commonly used term in the title or the abstract.	1	Abstract: "This retrospective single-center study…"; Materials and Methods: "This retrospective cohort study…"
1b	Provide in the abstract an informative and balanced summary of what was done and what was found.	1	Abstract: Background/Objectives, Methods, Results, and Conclusions
2	Explain the scientific background and rationale for the investigation being reported.	2-3	Introduction, paragraphs 1-3: rationale concerning postoperative wound complications, host-related biological factors, routine biomarkers, and the peri-ankle clinical model.
3	State specific objectives, including any prespecified hypotheses.	3	Introduction, final paragraph
4	Present key elements of study design early in the paper.	3-4	Materials and Methods, Sections 2.1 and 2.2: retrospective cohort design, patient selection, study period, clinically restricted cohort, and standardized surgical/perioperative management.
5	Describe the setting, locations, and relevant dates, including periods of recruitment, exposure, follow-up, and data collection.	3-5	Section 2.1: consecutive adult patients treated in the predefined date, single-center retrospective review at a tertiary trauma center. Section 2.2: standardized management and routine follow-up at 3 weeks, 6 weeks, 3 months, 6 months, and 1 year. Primary outcome window: first 3 postoperative weeks.
6a	Give the eligibility criteria, and the sources and methods of selection of participants. Describe methods of follow-up.	3-5	Section 2.1: consecutive adult patients with defined peri-ankle fractures; ORIF through a single open approach; detailed inclusion/exclusion criteria; 130 surgically treated patients, 72 excluded, 58 final cohort. Section 2.2: standardized inpatient and outpatient follow-up.
6b	For matched studies, give matching criteria and number of exposed and unexposed.	N/A	No matching was performed.
7	Clearly define all outcomes, exposures, predictors, potential confounders, and effect modifiers. Give diagnostic criteria, if applicable.	5-6	Sections 2.3-2.5 define postoperative WoC, perioperative biomarkers, composite biomarkers (NLR, SII, PNI, HALP), demographic variables
8	For each variable of interest, give sources of data and details of methods of assessment. Describe comparability of assessment methods if there is more than one group.	6-7	Section 2.4: laboratory data retrieved from the institutional electronic medical record, predefined sampling windows and selection of the initial available result.
9	Describe any efforts to address potential sources of bias.	3-7	Consecutive patient inclusion, predefined eligibility/exclusion criteria, restriction to a single calendar year and center, standardized surgical/perioperative management, predefined outcome window, routine follow-up, and explicit assessment of potential residual confounding and model overfitting.
10	Explain how the study size was arrived at.	3-4,7	Section 2.1: all consecutive eligible patients during the study period were included; 130 underwent surgery, 72 were excluded, and 58 constituted the final cohort. No additional sampling was performed and no formal a priori sample-size calculation was reported.
11	Explain how quantitative variables were handled in the analyses. If applicable, describe which groupings were chosen and why.	4-6	Sections 2.4-2.5: laboratory parameters were analyzed as continuous measures; continuous variables were summarized as median (IQR) and minimum–maximum. No categorization was used in the regression analyses. ROC cutoffs were data-derived using the Youden index.
12a	Describe all statistical methods, including those used to control for confounding.	7-8	Section 2.5: Kolmogorov–Smirnov test and histograms; Mann–Whitney U; chi-square/Fisher exact tests; BH-FDR sensitivity analyses; ROC analyses; separate preoperative and early postoperative multivariable logistic regression models; review of overlapping variables and restriction of covariates to reduce overfitting.
12b	Describe any methods used to examine subgroups and interactions.	N/A	No subgroup or interaction analyses were performed because of the limited sample size and outcome events.
12c	Explain how missing data were addressed.	3-4	Section 2.1: patients with incomplete perioperative laboratory or postoperative follow-up data were excluded; the final analytic cohort contained complete data for the variables included in the analyses.
12d	Cohort study-If applicable, explain how loss to follow-up was addressed.	N/A	No loss-to-follow-up analysis was required for the primary 3-week outcome window; patients without sufficient postoperative follow-up for wound assessment were excluded.
12e	Describe any sensitivity analyses.	6-7; Results, Tables 1-2	Section 2.5: BH-FDR sensitivity analyses were performed separately for the preoperative and early postoperative biomarker sets. FDR adjustment was not used as a variable-selection criterion for multivariable models.
13a	Report numbers of individuals at each stage of study.	3-4,7	Figure 1 and Section 2.1: 130 surgically treated patients during the study period; 72 excluded according to predefined criteria; 58 included in the final cohort; 18 developed WoC and 40 did not.
13b	Give reasons for non-participation at each stage.	7	Figure 1: exclusion categories and numbers are presented for patients excluded from the initial surgically treated population.
13c	Consider use of a flow diagram.	7	Figure 1: flow diagram of patient selection, exclusion criteria, and fracture diagnosis distribution in the final cohort.
14a	Give characteristics of study participants and information on exposures and potential confounders.	7-10	Results and Tables 1-2: age, sex, preoperative and early postoperative laboratory biomarkers, and composite inflammatory/nutritional indices are reported by WoC status.
14b	Indicate number of participants with missing data for each variable of interest.	3-4,7, Figure	Section 2.1 states that patients with incomplete relevant data were excluded, Figure 1: exclusion categories and numbers are presented for patients excluded from the study

14c	Cohort study-Summarize follow-up time.	4-6	Section 2.2-2.3: routine outpatient follow-up was scheduled at 3 weeks, 6 weeks, 3 months, 6 months, and 1 year; the primary outcome observation window was the first 3 postoperative weeks.
15	Cohort study-Report numbers of outcome events or summary measures over time.	7-10	Results: 18/58 patients (31.0%) developed postoperative WoC and 40/58 (69.0%) had uncomplicated wound healing. The primary outcome was assessed during the first 3 postoperative weeks.
16a	Give unadjusted estimates and, if applicable, confounder-adjusted estimates and their precision. Make clear which confounders were adjusted for and why they were included.	8-11	Tables 1-5 and Results: univariate comparisons, FDR-adjusted P values, and separate multivariable logistic regression models with adjusted ORs and 95% CIs. Candidate variables were initially selected by nominal univariate significance; overlapping biological information and model complexity were reviewed before model construction.
16b	Report category boundaries when continuous variables were categorized.	N/A	No quantitative variables were categorized for the multivariable analyses. ROC cutoffs were derived using the Youden index and are explicitly described as exploratory data-derived thresholds.
16c	If relevant, consider translating estimates of relative risk into absolute risk for a meaningful time period.	N/A	Not applicable to the analyses reported.
17	Report other analyses done-e.g., analyses of subgroups and interactions, and sensitivity analyses.	8-11	Correlation analyses, BH-FDR sensitivity analyses, ROC analyses restricted to FDR-significant biomarkers, and separate preoperative and early postoperative multivariable logistic regression analyses are reported.
18	Summarise key results with reference to study objectives.	12	Discussion, first paragraph: principal findings are summarized in relation to the study objective, including preoperative sodium/total protein, early postoperative sodium, and exploratory PNI findings.
19	Discuss limitations of the study, taking into account sources of potential bias or imprecision. Discuss both direction and magnitude of any potential bias.	15-16	Discussion, Limitations: retrospective single-center design, small sample and 18 outcome events, overfitting/sparse-data concerns, residual confounding, strict exclusions and limited generalizability, fracture heterogeneity, unavailable biomarkers and perioperative variables, unavailable menopausal status, and incomplete capture of later wound events.
20	Give a cautious overall interpretation of results considering objectives, limitations, multiplicity of analyses, results from similar studies, and other relevant evidence.	12-16	Discussion: findings are explicitly interpreted as exploratory/hypothesis-generating; FDR sensitivity results, same-cohort ROC derivation/evaluation, residual confounding, small sample, and relevant literature are considered. Clinical utility is stated as not established.
21	Discuss the generalisability (external validity) of the study results.	15-16	Limitations and Generalisability discussion: the clinically restricted cohort and strict exclusions reduce heterogeneity but limit applicability to more heterogeneous trauma populations; larger prospective cohorts are recommended.
22	Give the source of funding and the role of the funders for the present study and, if applicable, for the original study on which the article is based.	16	Funding: “This research received no external funding.”
